# Independent and Interactive Effects of Genetic Background and Sex on Tissue Metabolomes of Adipose, Skeletal Muscle, and Liver in Mice

**DOI:** 10.3390/metabo12040337

**Published:** 2022-04-08

**Authors:** Ann E. Wells, William T. Barrington, Stephen Dearth, Nikhil Milind, Gregory W. Carter, David W. Threadgill, Shawn R. Campagna, Brynn H. Voy

**Affiliations:** 1 UT-ORNL Graduate School of Genome Science and Technology, University of Tennessee-Knoxville, Knoxville, TN 37996, USA; ann.wells@jax.org; 2 The Jackson Laboratory, 600 Main Street, Bar Harbor, ME 04609, USA; nmilind2@ncsu.edu (N.M.); gregory.carter@jax.org (G.W.C.); 3 Department of Molecular and Cellular Medicine, Texas A&M Health Science Center, College Station, TX 77843, USA; wbarrington10@gmail.com (W.T.B.); dwthreadgill@tamu.edu (D.W.T.); 4 Department of Chemistry, University of Tennessee-Knoxville, Knoxville, TN 37996, USA; sdearth@vols.utk.edu (S.D.); campagna@utk.edu (S.R.C.); 5 Biological and Small Molecule Mass Spectrometry Core, University of Tennessee-Knoxville, Knoxville, TN 37996, USA; 6 Department of Animal Science, University of Tennessee-Knoxville, Knoxville, TN 37996, USA

**Keywords:** metabolomics, standard chow, metabolite, A/J, C57BL/6J, FVB/NJ, NOD/ShiLtJ, LC/MS, mice, male, female, diet, strain, genetics, sex, sex-by-strain

## Abstract

Genetics play an important role in the development of metabolic diseases. However, the relative influence of genetic variation on metabolism is not well defined, particularly in tissues, where metabolic dysfunction that leads to disease occurs. We used inbred strains of laboratory mice to evaluate the impact of genetic variation on the metabolomes of tissues that play central roles in metabolic diseases. We chose a set of four common inbred strains that have different levels of susceptibility to obesity, insulin resistance, and other common metabolic disorders. At the ages used, and under standard husbandry conditions, these lines are not overtly diseased. Using global metabolomics profiling, we evaluated water-soluble metabolites in liver, skeletal muscle, and adipose from A/J, C57BL/6J, FVB/NJ, and NOD/ShiLtJ mice fed a standard mouse chow diet. We included both males and females to assess the relative influence of strain, sex, and strain-by-sex interactions on metabolomes. The mice were also phenotyped for systems level traits related to metabolism and energy expenditure. Strain explained more variation in the metabolite profile than did sex or its interaction with strain across each of the tissues, especially in liver. Purine and pyrimidine metabolism and pathways related to amino acid metabolism were identified as pathways that discriminated strains across all three tissues. Based on the results from ANOVA, sex and sex-by-strain interaction had modest influence on metabolomes relative to strain, suggesting that the tissue metabolome remains largely stable across sexes consuming the same diet. Our data indicate that genetic variation exerts a fundamental influence on tissue metabolism.

## 1. Introduction

Metabolic phenotypes such as body composition and energy expenditure vary widely among individuals within a population. This variation contributes to the ability to maintain a healthy body weight, and to the long-term risk of metabolic diseases such as obesity and type 2 diabetes (T2D). Genetic background is a major contributor to inter-individual differences in metabolic traits. For example, large-scale studies of twins estimate the genetic contribution to variation in body mass index, which is widely used to assess obesity, to be as high as 85% [1].

Fundamental differences in metabolism at the cellular level contribute to variation in metabolic phenotypes between individuals. Metabolomics has emerged as a valuable platform for querying cellular metabolism and efficiently quantifying large sets of metabolites in experimental and clinical samples. Some classes of metabolites are thought to be highly heritable [2,3]. Accordingly, specific metabolite profiles have been linked to genetic susceptibility to disease studies in large-scale GWAS studies [4,5]. Nonetheless, population structure, rare variants, and non-genetic, unmeasured differences in diet, environment, and lifestyle complicate efforts to delineate the influence of genetic background on metabolomes in humans [6]. Understanding how genetic background influences metabolic profiles is important for precision medicine and for tailoring pharmacological treatments to the individual [2]. This insight is particularly relevant for developing strategies to improve metabolic health through precision nutrition and for targeted diet to optimize metabolic health [7].

Inbred strains of laboratory mice are a valuable set of models with which to understand the influence of genetic background on metabolism. Inbreeding allows genetic replicability, and laboratory husbandry provides the ability to control the diet and environment. Although mice and humans differ, many aspects of metabolism are shared between the two species. In addition, mice allow metabolomes to be profiled at the tissue level, where the underlying inputs to organismal level metabolic differences arise. Here, we utilize this control to evaluate the impact of both genetic background and sex on tissue metabolomes. We used untargeted metabolomics to quantify metabolites in three key metabolic tissues (adipose, liver, and skeletal muscle) from four common inbred strains of mice (A/J, C57BL/6J, FVB/NJ, and NOD/ShiLtJ) maintained under the same diet and husbandry conditions, and of the same age. Like individuals in a population, these strains represent varying levels of adiposity, metabolic health, and response to common human-relevant diets, and the variation in these traits differs between males and females. For example, A/J mice are relatively resistant to diabetes and obesity, while C57BL/6J mice are susceptible to these same disorders [8,9,10,11]. NOD/ShiLtJ mice are prone to developing type 1 diabetes [12]. FVB/NJ mice have high rates of activity, but are genetically prone to certain cancers and to anxiety [13,14]. Male and female mice were phenotyped for metabolic rate and their body composition and metabotyped for the effects of strain, sex, and their interactions. Tissue metabolite profiles were associated with system level markers of energy expenditure and body composition. Collectively, we report that genetic background has a major influence on tissue metabolomes.

## 2. Results

### 2.1. Physiological Measurements Reveal Differences among Strain and Sex-by-Strain

**Figure 1:**
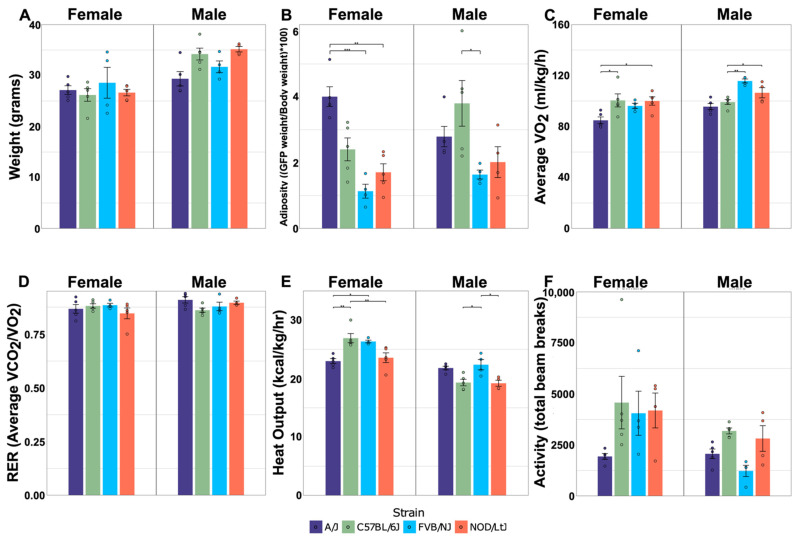
**Effects of sex and strain on metabolic traits.** Body weight (**A**) and adiposity (**B**) were measured at 28 weeks of age. VO_2_ (**C**), RER (**D**), heat output (**E**), and activity (**F**) were measured at 18 weeks of age, during a 48-h period when mice were housed in Phenomaster metabolic cages; N = 5/sex and strain group, avg. ± std. dev. Dots represent individual mice within sex-by-strain combination. Horizontal bars represent pairwise comparisons performed using Tukey’s HSD post hoc analysis; * *p* < 0.05, ** *p* < 0.01, and *** *p* < 0.001.

Body composition and indirect calorimetry were used to quantify the effects of sex and strain on key system-level metabolic phenotypes. Males were heavier than females, regardless of strain (Figure 1A). Body weight did not differ significantly between strains, and relatively modest effects of an interaction between sex and strain (*p* = 0.052) were observed for weight (Table 1). The relative weight of adipose tissue (adiposity) varied significantly between strains in a manner that was influenced by sex. For example, A/J mice were the fattest of the females, with adiposity levels approximately twice those of the other three strains, while C57Bl/6J was the fattest strain for male mice (Figure 1B). Interactions between sex and strain also significantly influenced oxygen consumption (VO_2_; *p* = 0.023) and heat output (*p* < 0.001) (Table 1). VO_2_, an indirect measurement of energy expenditure, was lowest in A/J mice of both sexes (Figure 1C). Heat output was also lowest in A/J mice among females (Figure 1E). C57Bl/6J females produced significantly more metabolic heat than the other strains, but heat production in C57Bl/6J was relatively low in male mice. Activity level was not influenced by the interaction between sex and strain, but was significantly affected by each parameter independently (Table 1). Females had significantly higher levels of activity, while C57BL/6J mice had the highest levels of activity relative to all other strains (Figure 1F). The respiratory exchange ratio was not significantly affected by sex, strain, or sex-by-strain, which was expected given that all strains were fed the same diet (Figure 1D). Collectively, these results confirm that both sex and genetic variation influence important physiological traits that are relevant to metabolism in this set of inbred strains.

### 2.2. Significant Effects of Strain, Sex and Their Interactions on Tissue Metabolomes

Liver, skeletal muscle, and adipose tissue are fundamental to systemic energy balance and metabolic health. Global metabolomic profiling of these tissues was used to characterize the effects of sex and strain on tissue metabolism. A total of 167 identified metabolites (those for which *m*/*z* and retention time have been matched to standards in our library) were detected in one or more tissues. In addition, several thousand spectral features corresponding to unidentified metabolites (4051, 3591, and 3228) were detected in liver, skeletal muscle, and adipose, respectively.

**Figure 2:**
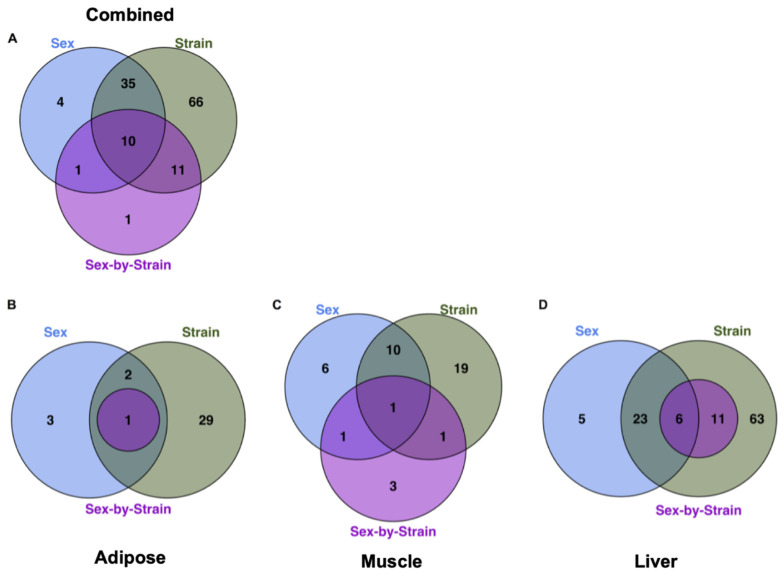
**Relative impact of sex, strain, and sex-by-strain interactions on tissue metabolomes.** (**A**) The number of metabolites that differed significantly by strain, based on ANOVA (FDR < 0.05), across tissues. The number of metabolites that differed significantly by sex, strain, and sex-by-strain interaction in (**B**) adipose, (**C**) muscle, and (**D**) liver.

**Figure 3:**
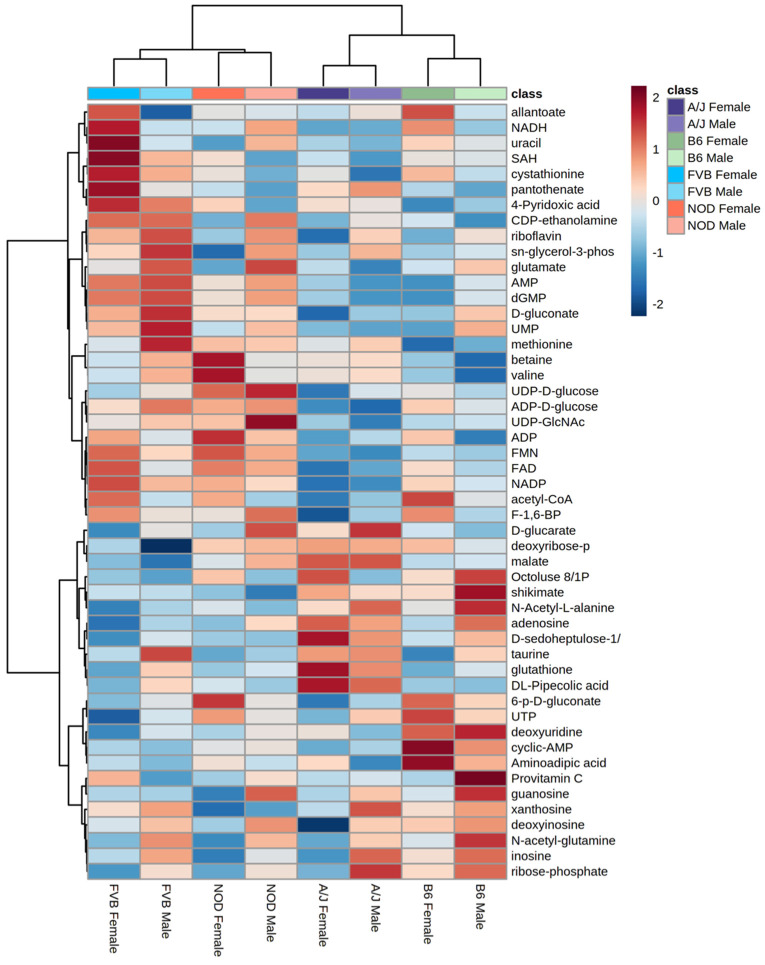
**Hierarchical clustering by sex and strain based on metabolite abundance in liver.** Heatmaps were generated in Metaboanalyst (v.5.0) using peak abundances of all metabolites. Hierarchical clustering was performed on group averages using Pearson distance and Ward clustering; scale is based on Z-score.

An ANOVA model was used to evaluate the relative effects of sex, strain, and sex-by-strain interactions on the metabolomes of each tissue. A total of 128 metabolites (78% of all identified metabolites) were significantly affected by one or more factors, in one or more tissues (Figure 2A), with approximately 95% (122 of 128) influenced by strain alone. Strain explained more variance in metabolite abundance than sex in each of the three tissues. For example, 31 metabolites differed according to strain in adipose tissue, while only 5 were affected by sex (Figure 2B). Similarly, 29 metabolites were affected by strain in muscle, with 16 differing between males and females (Figure 2C). In each tissue, the relative impact of the interaction between strain and sex was less than the influence of either factor alone. For example, 91 metabolites differed significantly by sex and/or strain in liver, but only 17 were significantly influenced by the interaction between sex and strain (Figure 2D). The metabolome of the liver showed more effects of both sex and strain than did adipose or muscle (Figure 2D). The unsupervised hierarchical clustering of the liver metabolites illustrates the relative impact of strain and sex (Figure 3). Based on metabolite abundance, the clusters formed based on strain and then sex (Figure 3). Collectively, these results indicate that genetic background had the predominant influence on the metabolome, regardless of tissue.

### 2.3. Partial Least Squares Discriminant Analysis Reveals Strain Is a Discriminant of Metabolites

**Figure 4:**
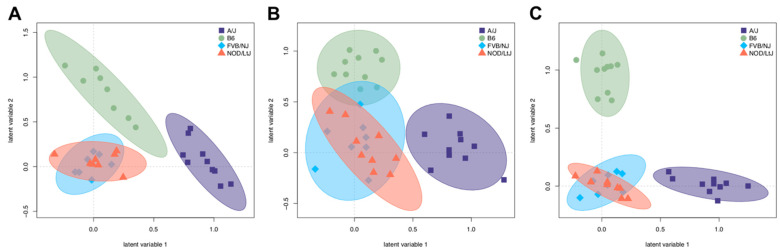
**Partial least squares discriminant analysis (PLS-DA) represents separation of strains based on tissue metabolomes.** Unknown and known metabolites were classified by A/J, C57BL/6J, FVB/NJ, and NOD/ShiLtJ in (**A**) adipose, (**B**) skeletal muscle, and (**C**) liver. The 95% CI was determined using Hotelling’s T^2^ ellipses.

Partial least squares discriminant analysis (PLS-DA) was performed using both identified and unidentified metabolites in each tissue to visualize the relative extent to which the metabolites discriminated the strains in each tissue (Figure 4). Both A/J and C57BL/6J mice separated from FVB/NJ and NOD/ShiLtJ, based on a lack of overlap of 95% confidence intervals, particularly in adipose (Figure 4A) and liver (Figure 4C), but not in muscle (Figure 4B). FVB/NJ and NOD/ShiLtJ mice were not readily separable from each other, which is consistent with their genetic relatedness compared to the other two strains [15]. The variable importance projections (VIP) for each tissue were identified to compare the sets of metabolites that discriminate strains in each tissue (Supplemental Tables S1–S3). Sixty-five, fifty-five, and sixty-nine known metabolites had a VIP > 1 in adipose, muscle, and liver, respectively. The overlap between these three subsets showed that 16, 28, and 35 metabolites were unique to adipose, muscle, and liver tissue, while 9 metabolites were identified in all three tissues. Metabolites that discriminate strains in all three tissues include intermediates of glycolysis (fructose 1,6-bisphosphate, 3-phosphoglycerate), amino acid synthesis (lysine, methionine, tyrosine), lysine catabolism (aminoadipate), and the tricarboxylic acid (TCA) cycle (fumarate). These results indicate that although metabolome profiles discriminate strains in a similar manner in each tissue, the specific sets of metabolites that drive separation tend to be tissue-specific.

### 2.4. Functional Annotation of Strain Effects

**Figure 5:**
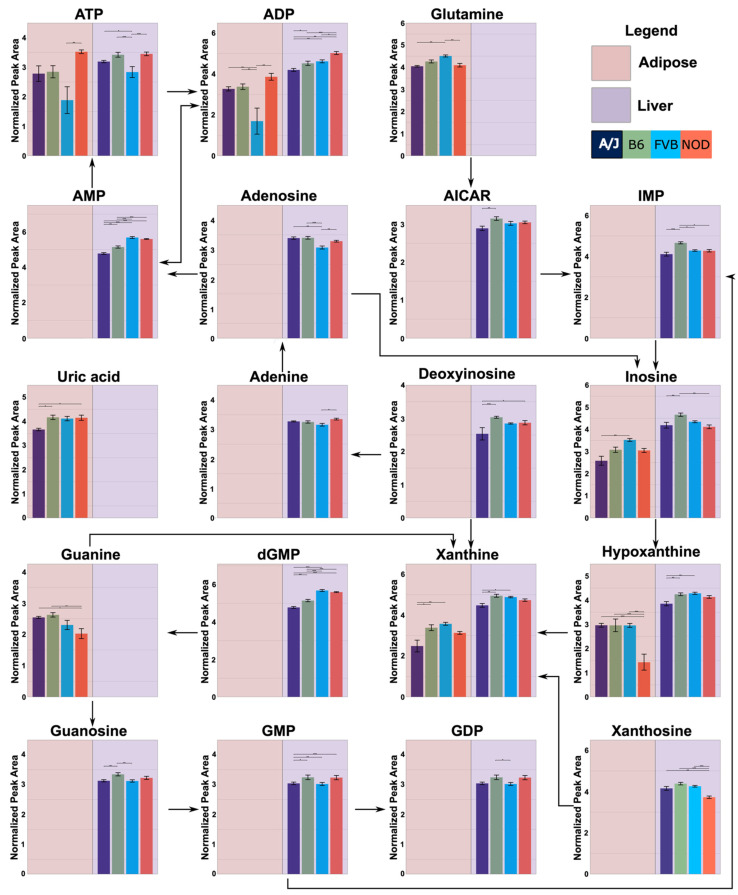
**Strain-specific differences in purine metabolite levels in adipose and liver tissue.** Metabolites that differed significantly between strains in adipose and/or liver were mapped onto the purine KEGG pathway. Significant differences between strains were determined using ANOVA and Tukey’s post hoc. Each bar plot was calculated by averaging the peak abundance of each mouse sample +/− standard error. The y-axis represents the abundance of the metabolite in arbitrary units. The x-axis represents each strain, which is color-coded and listed in the legend. Connections between metabolites indicated by arrows are based on the purine KEGG pathway. Dashed lines represent missing metabolites in the pathway. Data for adipose or liver are only shown if strain significantly affected metabolite abundance. Horizontal bars represent pairwise comparisons performed using Tukey’s HSD post hoc analysis; * *p* < 0.05, ** *p* < 0.01, and *** *p* < 0.001.

Functional annotation based on the Kyoto Encyclopedia of Genes and Genomes (KEGG) pathway enrichment was used to characterize the effects of strain, as well as sex and sex-by-strain interaction, on tissue metabolomes (Table 2). The combined set of metabolites that differed significantly between strains in one or more tissues was enriched in components of purine and pyrimidine metabolism. These relationships were largely due to the effects of strain in liver and adipose (Figure 5), where 30 and 13 metabolites, respectively, mapped onto these two KEGG pathways. Purine metabolites were also influenced by sex in liver (*n* = 9), although not in adipose or muscle. Metabolites that differed between strains in adipose tissue were also involved in amino acid (arginine and proline) metabolism (*n* = 5) and aminoacyl-tRNA-biosynthesis (*n* = 6), while the pathway for pantothenate and CoA biosynthesis (*n* = 5) was affected by strain in liver. In muscle, the sets of metabolites that differed significantly between strains and between sexes were enriched for components of protein synthesis (aminoacyl-tRNA-biosynthesis (*n* = 6)). These results highlight purine and pyrimidine metabolism, particularly in liver and adipose tissue, as pathways that may contribute to the effects of genetic background on overlying systems level metabolic phenotypes.

Assessing intermediary genes, using the mouse phenome database, between the metabolites identified in purine metabolism revealed potential single nucleotide polymorphisms (SNPs) that may contribute to metabolite abundance between strains (Table 3). These SNPs could alter gene function, affecting the gene’s ability to convert metabolites within the pathway, ultimately affecting metabolite abundance. Furthermore, the assessment of pairwise comparisons using Tukey’s post hoc analysis showed that each metabolite identified had different relative abundances between strains, indicating a complex relationship between genetics and metabolite levels throughout the metabolic pathways.

### 2.5. Connecting Metabolic Profiles to Traits

**Figure 6:**
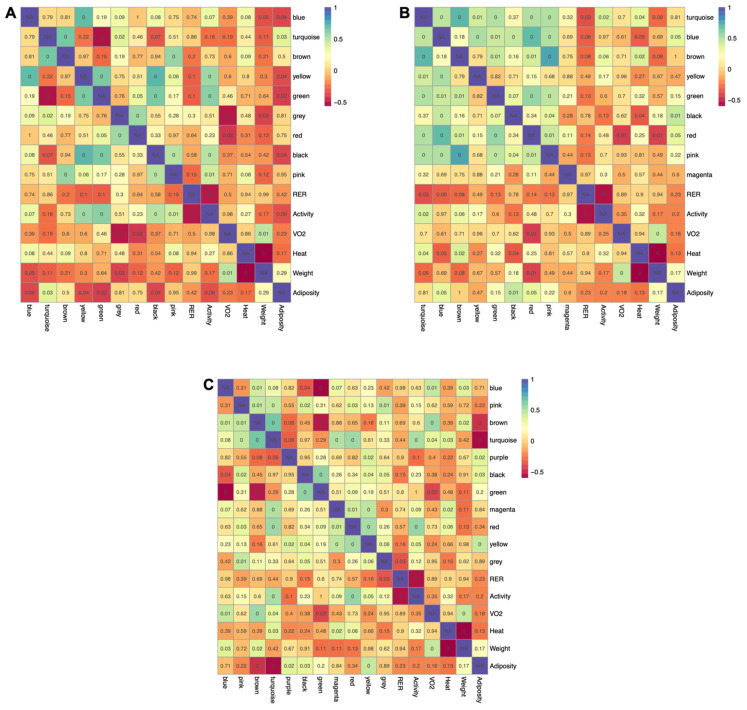
**Relationship between modules and physiological measurements for each tissue using Pearson correlation.** Correlation heatmaps for adipose (**A**), muscle (**B**), and liver (**C**) depict positive and negative relationships, with the *p*-value of each relationship overlayed onto the heatmap and corrected using the Benjamini–Hochberg procedure.

Metabolic pathways are intertwined, and the levels of individual metabolites within a given pathway are highly interdependent. To incorporate this behavior into the analyses, weighted gene co-expression analysis (WGCNA) was implemented to identify clusters of highly intercorrelated metabolites (modules) within each tissue. WGCNA was developed to extract co-expressed sets of genes from transcriptomic datasets, but more recently has been adopted for use with metabolomics data. Using this approach, a signed and weighted network was constructed from metabolite abundance based on correlations, and sets of highly interconnected metabolites were represented as modules. Modules were then associated with phenotypes based on the correlation of the module’s representative eigenmetabolite with each physiological trait. There were nine, nine, and eleven modules identified within adipose, muscle, and liver, respectively (Supplemental Figure S3). Five of the nine modules extracted from adipose tissue (yellow, turquoise, green, blue, and black) were significantly correlated with adiposity, with four having inverse relationships (Figure 6A). Four modules (yellow, pink, green, and black) were positively correlated with activity level. Interestingly, three of these same modules (yellow, green, and black) were inversely related to adiposity, suggesting that the reciprocal relationship between energy expenditure and fat accretion are reflected at the metabolite level in adipose tissue. Similar relationships were observed in muscle (Figure 6B), with the blue and black modules positively related to adiposity and inversely correlated to metabolic heat. Two modules found in muscle (turquoise and brown) were positively correlated with both activity and heat, reflecting the interrelationship between these traits. Four modules in liver (Figure 6C) were associated with adiposity (two positively and two inversely), one of which (turquoise) was also positively correlated with activity, heat, and VO_2_. This module was inversely related to adiposity, further indicating that metabolite abundance in tissues reflects the interrelationships between higher order metabolic traits.

## 3. Discussion

Genetic background influences metabolic health, but the relationship between genetics and metabolism at the tissue level is poorly understood. Metabolites are a product of cellular processes in the body and are therefore thought to be most closely related to phenotypes. We used untargeted metabolomics to investigate the effects of strain and sex on the metabolomes of adipose tissue, liver, and skeletal muscle because of their central roles in metabolism and energy expenditure. We also associated metabolites to body composition and energy expenditure. Similar to individuals within a population, the mouse strains chosen vary in terms of genetic backgrounds and potential for metabolic disease, and in ways that differ between males and females.

We found that genetic background had a significant effect on the tissue metabolite profiles, regardless of the tissue that was profiled. The majority of known metabolites detected on our platform were significantly affected by strain in at least one tissue, based on the ANOVA results. Likewise, PLS-DA discriminated strains based on metabolite profiles, with A/J and C57BL/6J each distinctly separated from FVB/N and NOD mice. Our findings support the concept of individual “metabotypes” (metabolic phenotypes), a term used to describe the variation in metabolite profiles that exists between individuals [16]. More specifically, our results indicate that genetic variation is likely a major determinant of metabotypes. Metabotypes have been shown to be surprisingly stable across various physiological states and over time [17]. In a longitudinal study of 818 individuals, 95% of participants showed a high degree (>70%) of metabotype conservation over a seven-year period [18]. Understanding the basis for a metabotype is highly relevant to the emerging fields of precision nutrition and personalized medicine, which seek to tailor diets or therapeutic interventions to the individual [19,20]. Distinct metabotypes detected in blood or urine from clinical populations have been associated with the incidence and progression of various diseases such as cardiovascular disease and diabetes [17,21]. In addition to disease risk, metabotypes have been associated with interindividual differences in fundamental physiological traits. Chu and coworkers recently applied the metabolome profiling of blood to a cohort of individuals that had been richly phenotyped for baseline immune parameters [5]. Distinct metabolite profiles were associated with various cytokine measures and explained up to 30% of the variation in cytokine responses that existed between individuals. Metabotypes have also been used to design diets that manipulate the post-prandial glycemic response to a meal in predictable directions, demonstrating the application of metabolic phenotypes to precision nutrition [7].

The integration of metabolomics in genome-wide association studies (GWAS) supports the concept that genetic background influences metabolite profiles. Loci linked to metabolite profiles (mQTLS) have been identified in numerous studies [5]. Further, a recent metanalysis of GWAS indicated that heritability estimates approach 50% for some classes of metabolites [3]. While these various studies point to a role for genetic background, it remains difficult to uncouple the impact of polymorphic variation from other non-genetic sources of variation that exist in humans [22]. Large-scale studies allow the confounding effects of known variation in diet, age, environment, and lifestyle to be incorporated into models. However, the impact of variation in the gut microbiota, which contributes metabolites to the circulatory system, is difficult to account for, but is likely to influence metabolite profiles in body fluids, which are typically the target of metabolome profiling in humans. In contrast, our data are derived directly from tissues, which are less likely to contain metabolites produced by the microbiota. In addition, the ability to profile multiple individuals within a strain provided us with a level of genetic replication that, outside of twin studies, is not available in human studies. Our experimental design also used mice of the same age, which allowed us to control for interactions between sex and age that have been described in other metabolomics studies [23].

Nearly a third (30 of 103) of metabolites in liver that were influenced by strain are involved in purine and pyrimidine metabolism. Purines and pyrimidines are central to the function of all cells as subunits of nucleic acids. In addition, these nucleotides and their derivatives carry energy, allosterically control enzymatic reactions, mediate intra- and intercellular signaling pathways, and participate in the control of cellular metabolism. Purine metabolism, in particular, is linked to pathways that govern cellular energy allocation through its relationship to AMPK (5′ AMP-activated protein kinase), a kinase that is allosterically activated by a rise in AMP:ATP. AMPK mediates the response to an energy deficit by activating the pathways that generate ATP and inhibiting those that consume ATP. AMPK can also be activated by the ZMP (5-aminoimidazole-4-carboxamide ribonucleotide), an intermediate in de novo purine synthesis that acts as an AMP mimetic. Recent studies have shown that altering purine metabolism in ways that increase ZMP activates AMPK and elicits the same types of metabolic changes, such as the increased oxidation of fatty acids, that result from an increase in AMP. Our assessment of SNPs identified several potential variants that may contribute to genetic differences in nucleotide metabolism between the inbred strains used here. In general, our data are consistent with a recent study of the tissue metabolomes of three inbred strains, in which purine metabolism was also identified as a pathway in the differentiated mice of different genetic backgrounds [12,24].

In conclusion, we have used the replicability of inbred strains and the control provided by laboratory husbandry to compare the effects of genetic background, sex, and their interactions on the metabolomes of key metabolic tissues. We provide evidence that genetic background, more so than sex, significantly influences tissue metabolomes, particularly in liver. Future studies are needed to determine whether and how these underlying differences in metabolism contribute to the risks of various metabolic diseases that are represented by this collection of inbred strains. Our data further support the concept of individual metabotypes, which has important implications for the emerging field of precision health.

## 4. Materials and Methods

### 4.1. Animals and Diets

All husbandry and experimental procedures were approved by the Institutional Animal Care and Use Committee of the University of North Carolina. Four-week-old C57BL/6J mice were purchased from The Jackson Laboratory (Bar Harbor, ME, USA). The mice were allowed to acclimate for 14 days and consumed a standard mouse chow (PicoLab Mouse Diet 20, LabDiet, St. Louis, MO, USA) during this period. At 42 days of age, five male and five female mice from each strain were switched to a marginally lower protein standard chow diet (19% protein) and fed ad libitum. The mice were maintained on a 12 h light/dark cycle throughout the study. At age 18 weeks, after 12 weeks on the diets, the mice were housed in Phenomaster metabolic chambers (TSE Systems, Inc., Chesterfield, MO, USA) for 48 h for the measurement of metabolic rate and activity. The chambers measured respiratory exchange rate (RER), volume of oxygen (VO_2_), and heat output via heat dissipation, and activity level by laser detection through the ActiMot2 module. Activity levels measured voluntary movement of the mouse in the x and y plane by quantifying horizontal and vertical beam breaks. The mice were euthanized at 28 weeks of age by CO_2_ asphyxiation. Perigonadal adipose tissue was dissected and weighed as a measure of adiposity. Samples of adipose tissue, the left lobe of the liver, and the vastus medialis, vastus lateralis, and rectus femoris muscle were snap-frozen in liquid nitrogen and stored at −80 °C for metabolomics analysis.

### 4.2. Metabolite Extraction from Tissues

The frozen tissue samples were pulverized under liquid nitrogen and approximately 0.025 g of tissue was added to a 2 mL Eppendorf tube containing pre-chilled methanol (1.3 mL). Samples were extracted using the method described in [25]. Internal standard (60 μL of a ^13^C-labeled *E. coli* metabolite pool) was added to each sample.

### 4.3. Liquid Chromatography Mass Spectrometry

The samples, kept at 4 °C, were placed in an autosampler tray. A total of 10 μL from each sample was injected through a Synergi 2.5 micron Hydro-RP 100, 100 × 2.00 mm LC column (Phenomenex, Torrance, CA, USA) kept at 25 °C. The mass spectrometer (MS) was run in full scan mode, with negative ionization mode, using a method adapted from Lu et al. [26]. The eluent entered the MS via an electrospray ionization source attached to a Thermo Scientific Exactive Plus Orbitrap MS (Waltham, MA, USA) through a 0.1 mm internal diameter fused silica capillary tube. The samples were run with a spray voltage of 3 kV. The nitrogen sheath gas was set to a flow rate of 10 psi, with a capillary temperature of 320 °C. The AGC target was set to 3 × 10^6^. The samples were analyzed with a resolution of 140,000. A scan window of 85 to 800 *m*/*z* (mass-to-charge) was used from 0 to 9 min, and a window of 110 to 1000 *m*/*z* from 9 to 25 min. Solvent A consisted of 97:3 water:methanol, 10 mM tributylamine, and 15 mM acetic acid. Solvent B was methanol. The gradient from 0 to 5 min was 0% Solvent B, from 5 to 13 min was 20% Solvent B, from 13 to 15.5 min was 55% Solvent B, from 15.5 to 19 min was 95% Solvent B, and from 19 to 25 min was 0% Solvent B, with a flow rate of 200 µL/min.

### 4.4. Generation of ^13^C-Labeled E. coli

*E. coli* metabolites were labeled with ^13^C and extracted using a method adapted from Bennett et al. [27,28] and described in [25].

### 4.5. Metabolomics Data Processing

Raw spectra files generated by Xcalibur were converted to mzML, an open-source format, using msConvert [29]. An open-source data analyzer for metabolomics, MAVEN [30,31] (Princeton University) was used to retention time correct the total ion chromatograms based on peaks picked from the extracted ion chromatograms for each sample. Metabolite identities were assigned by matching mass-to-charge (*m*/*z*) (within ±5 ppm) and retention time (r.t.) (within ±30 s) to values measured from purified standard compounds that were used to construct a database for validated metabolites. The peak quality for all identified metabolites was checked manually, and metabolite abundance was quantitated as ion counts that were integrated from the area under each peak. Unidentified metabolites were chosen based on a peak-picking algorithm provided in MAVEN. These compounds were named as *m/z*:r.t. pairs, and the peak area was then integrated as described for the identified compounds using an algorithm described in [25]. Three samples (one each: FVB female, FVB male, NOD female) were removed from further analyses due to poor peak quality. The final sample sizes for metabolomics analyses were N = 5 for both sexes of A/J and C57, and for NOD males, with N = 4 for FVB (male and female) and for NOD male.

### 4.6. Use of ^13^C-Labelled E. coli Cellular Extracts as an Internal Standard

Prior to the use of diluted ^13^C-labeled *E. coli* as internal standards in the samples, triplicate aliquots of the labeled extracts were analyzed using the LC–MS metabolomics method [26]. The resulting data were then processed identically to the samples. Since the ^13^C-labeled *E. coli* internal standard was used as a normalization technique, verification that non-significant amounts of ^12^C-label remained within the samples was performed for the identified metabolites. Unlabeled metabolites were matched to their corresponding ^13^C-labeled internal standard if detected, or to a ^13^C-labeled standard of the same compound class if no stable isotope-labeled standard was detected (e.g., an amino acid with no exact standard match would be matched to a ^13^C-labeled metabolite with class type amino acid). The same ^13^C-labeled metabolite for a particular class type was used for the normalization of all metabolites of the same class type missing an exact ^13^C-label metabolite match (i.e., ^13^C-labeled tyrosine was used for all amino acids missing a ^13^C-labeled match). Class types were identified using the Human Metabolome Database (HMDB) [32,33,34] using the class types listed under each metabolite identified in the HMDB. The ion intensities for each unlabeled metabolite were normalized to those of the appropriate ^13^C-labeled standard in order to correct for instrumental variability among the sample analyses.

### 4.7. Statistical Analysis

All statistical analyses were performed in the language R (3.1.0 and 3.2.2) [35]. An ANOVA model was used to identify the significant effects of diet, sex, and sex-by-diet interaction on physiological traits. A significant F-test for the effects of strain, sex, or their interaction was followed by a Tukey’s Honest Significant Difference (HSD) test, used for post hoc testing. The significance for physiological traits was based on raw *p*-values (*p* < 0.05).

The same ANOVA model was used to identify the significant effects of strain, sex, and sex-by-strain interaction on metabolites, controlling for false discovery rate (FDR) to account for multiple testing (described in further detail below). Metabolite peak area data files were read into R using the package XLConnect [36]. Metabolites that were missing more than 10% of their sample measurements were removed from the analysis in order to obtain the most complete dataset possible. Missing values in the remaining metabolites were imputed using k-nearest numbers (k = 10) from the function impute [37]. Prior to the statistical analyses, a linear model was created for each metabolite using the terms sex, strain, sex*strain, tissue weight, and internal standard (Equation (1)):

Metabolite = sex + strain + sex*strain + tissue weight + internal standard(1)

This linear model was performed prior to formal statistical analysis to determine the coefficients for tissue weight and internal standard, which were utilized in Equation (2) to obtain metabolite abundances adjusted for tissue weight and internal standard.

Coefficients for the terms for tissue weight and internal standard were used to adjust metabolite abundance for technical variation using Equation (2):

Adjusted metabolite = metabolite + tissue weight coefficient (mean tissue weight − tissue weight) + internal standard coefficient (mean internal standard − internal standard)(2)

Adjusted metabolites were pareto scaled across all mice for each metabolite using the package MetabolAnalyze [38], normalized to the median across all metabolites for each mouse, and cube root transformed. Normalized metabolite values were analyzed for effects of sex, strain, and sex*strain. Data were assessed for normality using Q-Q plots, residuals, and the Shapiro–Wilk test. The false discovery rate for ANOVA and correlation analyses was set to 5% using the method of Benjamin–Hochberg [39]. FDR-adjusted *p*-values were estimated by calculating the q-value. Venn diagrams were created using the package VennDiagram [40].

### 4.8. Partial Least Squares Discriminant Analysis

Partial least squares discriminant analysis (PLS-DA) was used to characterize the strain, sex, and sex-by-strain effects on the tissue metabolome using identified and unidentified metabolites. The PLS-DA algorithm was implemented using the package DiscriMiner [41]. Scores plots with Hotelling’s T^2^ ellipses were created using the packages plyr and Car [42,43]. External cross-validation was used to assess the model performance and to reduce overfitting using 1000 permutations (Supplemental Figure S1). Two-thirds of the mice were randomly assigned as the training set, while the remaining mice were used as a test set, to determine the model performance. The assessment of the model was based on the model’s ability to correctly identify the treatment assignment of the new mice in the test set, based on the mice from the beginning training set.

### 4.9. Correlation Analysis

Associations between the physiological measurements and the metabolite measurements across tissues were assessed using Pearson correlation from the package Hmisc [44] and the package R.Utils [45]. Correlation *p*-values were FDR-adjusted using the Benjamini–Hochberg procedure. Correlations were visualized using the Cytoscape app MetScape [46].

### 4.10. Functional Pathway Analysis

Overrepresentation pathway analysis for the identified metabolites was performed using MetaboAnalyst [47]. KEGG IDs were input and compared against the Mus musculus KEGG reference metabolome. The statistical significance of pathway overrepresentation was evaluated using a Fisher’s exact test. The *p*-values were adjusted using the Benjamini–Hochberg procedure. Pathway topology was performed using relative-between centrality.

### 4.11. Weighted Gene Co-Expression Network Analysis

To identify potential metabolite networks, weighted gene co-expression network analysis (WGCNA) was performed using the ‘WGCNA’ R package [48]. The soft-thresholding power was chosen so that the scale-free topology correlation hits 9. Soft-thresholding powers chosen for adipose, muscle, and liver were 9, 8, and 5, respectively (Supplemental Figure S2). Dynamic tree cutting was used to cluster the metabolites and generate modules with a minimum of five metabolites in each module. Networks were identified using identified metabolites.

ANOVA was performed for each module using the linear model:

Module = sex + strain + sex*strain(3)

to determine the effects of the sex, strain, or sex-by-strain interaction on each module.

## Figures and Tables

**Table 1:** *p*-values for effects of sex, strain, and sex-by-strain interaction on weight and metabolism.

	Sex	Strain	Sex-by-Strain
**Weight**	**<0.001**	0.284	0.052
**Adiposity**	0.338	**<0.001**	**0.015**
**VO_2_**	**0.001**	**<0.001**	**0.023**
**RER**	0.148	0.594	0.100
**Heat Output**	**<0.001**	**0.001**	**<0.001**
**Activity**	**0.009**	**0.020**	0.124

*p* < 0.05 is considered significant.

**Table 2:** KEGG pathway enrichment of metabolites affected by sex, strain, and sex-by-strain.

Tissue	Factor	Pathway	Hits	*p*-Value	(i/m)q
**All Tissues**	**Sex**	Purine metabolism	9	<0.001	0.001
	**Strain**	Purine metabolism	20	<0.001	0.008
		Pyrimidine metabolism	15	<0.001	0.008
		Alanine, aspartate and glutamate metabolism	8	<0.001	0.008
		Ascorbate and aldarate metabolism	5	<0.001	0.008
		Citrate cycle (TCA cycle)	6	0.002	0.008
		Pantothenate and CoA biosynthesis	5	0.003	0.008
		Aminoacyl-tRNA biosynthesis	12	0.003	0.008
**Adipose**	**Strain**	Purine metabolism	8	<0.001	0.007
		Pyrimidine metabolism	5	0.001	0.007
		Arginine and proline metabolism	5	0.002	0.007
		Aminoacyl-tRNA biosynthesis	6	0.002	0.007
**Muscle**	**Sex**	Aminoacyl-tRNA biosynthesis	5	0.001	0.002
	**Strain**	Aminoacyl-tRNA biosynthesis	6	0.001	0.004
**Liver**	**Sex**	Purine metabolism	9	<0.001	0.003
	**Strain**	Purine metabolism	17	<0.001	0.003
		Pyrimidine metabolism	13	<0.001	0.003
		Pantothenate and CoA biosynthesis	5	0.002	0.003
	**Sex-by-Strain**	Pyrimidine metabolism	4	0.001	0.002

**Table 3:** SNPs across mouse strains reveal genetic variation.

		SNP Type per Mouse Strain			
Intermediary Gene	Pathway Step	A/J	C57BL/6J	FVB/NJ	NOD/ShiLtJ
Enpp1	ATP → AMP	Cn, Cs		Cn, Cs	U3, Cn, Cs
Ak7	AMP → ADP				U3, Cn, Cs
Gdr	Guanine → Xanthine	U3		U3	
Nt5c3b	Adenosine → Inosine	Cn, Cs			
Xdh	Xanthine → Uric acid				U3, Cn, Cs
Guk1	GMP → GDP	U3, U5, Cn		U3, U5, Cn	U3, U5, Cn

Potential SNPs were identified using the Mouse Phenome Database SNP data retrieval utility (phenom.jax.org, accessed on 22 February 2022). All SNPs are relative to C57BL/6J mice. Cn-non-synonymous SNP, Cs-synonymous SNP, U5-UTR variant of the 5′ UTR, U3-UTR variant of the 3′ UTR.

## Data Availability

The data presented in this study are available on request from the corresponding author. The data are not publicly available due to privacy.

## Supplementary Materials

The following are available online at https://www.mdpi.com/article/10.3390/metabo12040337/s1, Figure S1: Density and frequency plots of PLS-DA permutations, Figure S2: Choosing soft-threshold power, Figure S3: Topological overlap matrix and module bar plots, Table S1: Variable importance projections for adipose partial least squares discriminant analysis (Excel file), Table S2: Variable importance projections for skeletal muscle partial least squares discriminant analysis (Excel file), Table S3: Variable importance projections for liver partial least squares discriminant analysis (Excel file).
