# Supplementary material for: Independent and Interactive Effects of Genetic Background and Sex on Tissue Metabolomes of Adipose, Skeletal Muscle, and Liver in Mice
Source: Metabolites. 2022 Apr 8;12(4):337. doi: 10.3390/metabo12040337 (PMC9031494; doi:10.3390/metabo12040337)
Supplement: Supplementary file 1 [file metabolites-12-00337-s001.zip › Supplemental Figures S1-S3.pdf]

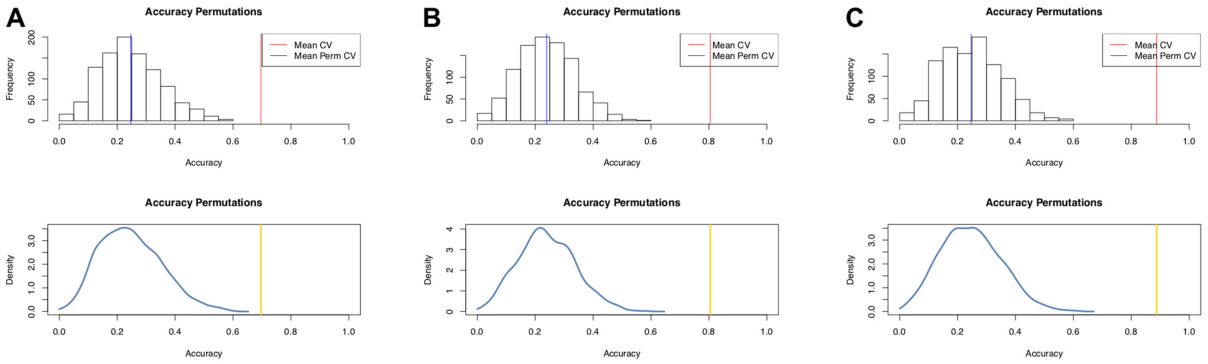

Supplemental Figure S1. Density and frequency plots of PLS-DA permutations.

1000 permutations were performed and plotted for adipose, $p < 0.001$ (**A**), muscle, $p < 0.001$ (**B**), and liver, $p < 0.001$ (**C**).

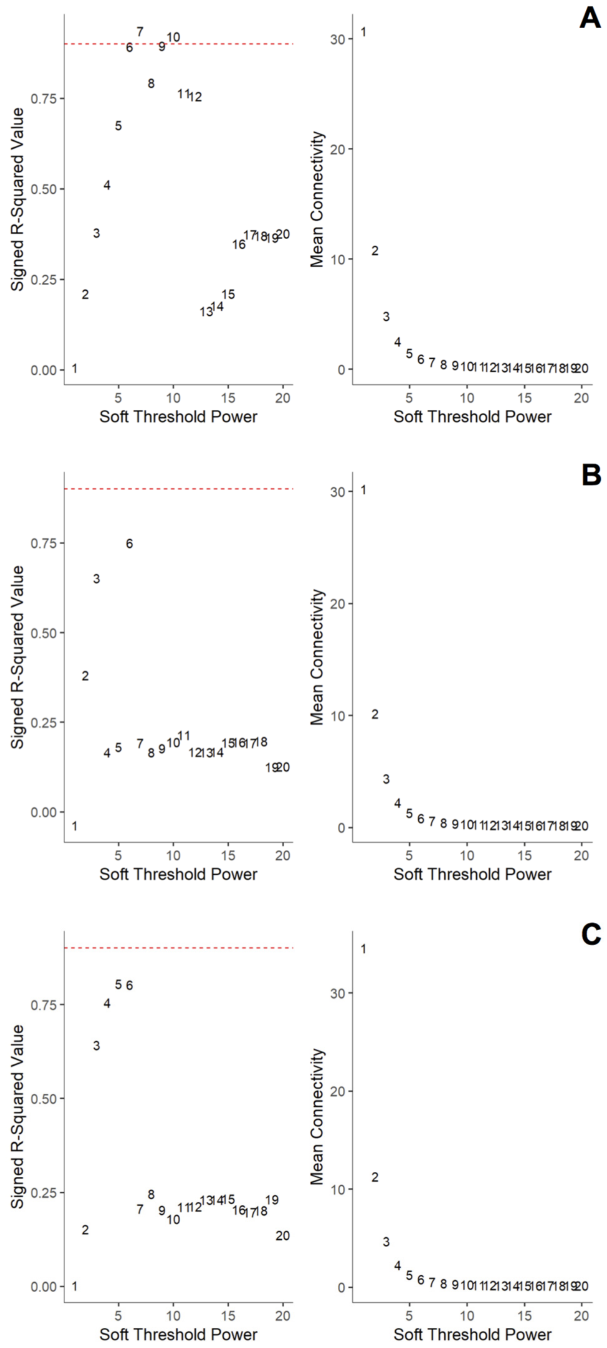

Supplemental Figure S2. Choosing soft threshold power. The smooth softmax function is used to create a scale-free topology on the metabolite abundance network. The aim is to reach an r-squared value of 0.9, however, if that is unattainable then the highest r-squared value is identified. The soft threshold power for adipose is 6 (**A**), muscle is 3 (**B**), and liver is 5 (**C**).

A

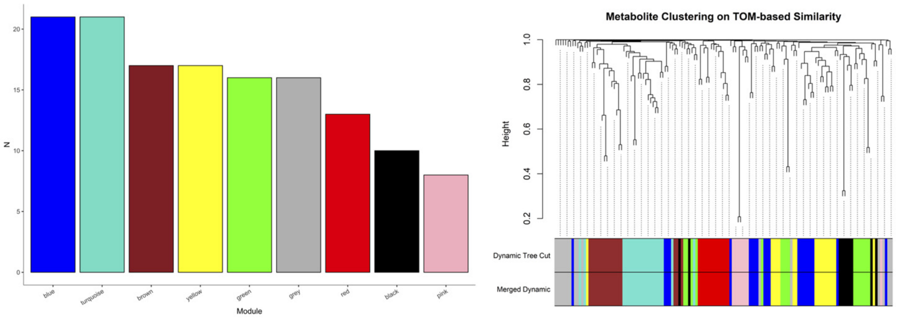

B

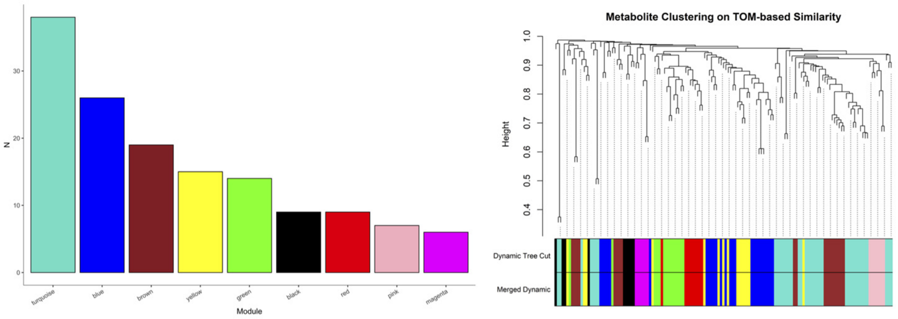

C

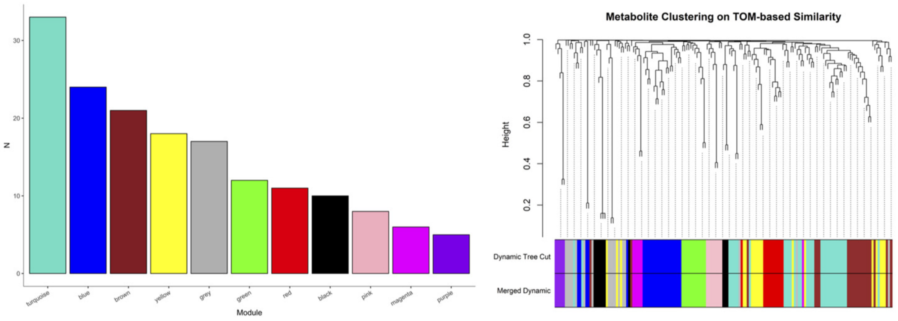

Supplemental Figure S3. Left: Topological Overlap Matrix (TOM). The TOM is created for the adjacency matrix to reduce noise and spurious associations. Modules that are then determined to be highly correlated (r > 0.75) are merged to reduce

redundancy. Merged modules were identified for adipose (**A**), muscle (**B**), and liver (**C**) tissue. **Right: Module Barplots.** The number of each metabolites present in each module, as well as the number of modules created for adipose (**A**), muscle (**B**), and liver (**C**) tissue.
